# Longitudinal follow-up study of the association between statin use and chronic periodontitis using national health screening cohort of Korean population

**DOI:** 10.1038/s41598-022-09540-y

**Published:** 2022-04-01

**Authors:** Mi Jung Kwon, Soo-Hwan Byun, Joo-Hee Kim, Ji Hee Kim, Se Hoon Kim, Nan Young Kim, Hye-Rim Park, Hyo Geun Choi

**Affiliations:** 1 Department of Pathology, Hallym University Sacred Heart Hospital, Hallym University College of Medicine, Anyang, Republic of Korea; 2 Department of Oral and Maxillofacial Surgery, Dentistry, Hallym University Sacred Heart Hospital, Hallym University College of Medicine, Anyang, Republic of Korea; 3 Division of Pulmonary, Allergy, and Critical Care Medicine, Department of Medicine, Hallym University Sacred Heart Hospital, Hallym University College of Medicine, Anyang, Republic of Korea; 4 Department of Neurosurgery, Hallym University Sacred Heart Hospital, Hallym University College of Medicine, Anyang, Republic of Korea; 5 Department of Pathology, Severance Hospital, Yonsei University College of Medicine, Seoul, Republic of Korea; 6 Hallym Institute of Translational Genomics and Bioinformatics, Hallym University Medical Center, Anyang, Republic of Korea; 7 Department of Otorhinolaryngology-Head and Neck Surgery, Hallym University Sacred Heart Hospital, Hallym University College of Medicine, 22, Gwanpyeong-ro 170 beon-gil, Dongan-gu, Anyang-si, Gyeonggi-do 14068 Republic of Korea

**Keywords:** Health care, Medical research, Risk factors

## Abstract

Since a potential link between statins and the risk of adverse chronic periodontitis (CP) has been raised, we aimed to validate the association between statin use and the incidence of CP using nationwide cohort data. This longitudinal follow-up study included 169,381 patients prescribed statins who were matched with an equal number of controls using propensity scores from the Korean National Health Insurance Service-Health Screening Cohort database (2002–2015). A Cox proportional hazard model was used to assess the occurrence of CP following statin use after adjusting for multiple covariates. The occurrence of CP was significantly higher in patients who had long-term use (1–3 years, 3–5 years, or > 5 years) than with short-term use (≤ 1 year) of statins. After adjustment, statin users exhibited an occurrence of CP 1.32-fold higher (95% confidence interval 1.30–1.33) than that of the matched nonusers (incidence: 25.0 and 22.0 per 100 person-years, respectively). Subgroup analyses supported the adverse impact of statins on CP independent of age and gender. Statin user odds ratios for developing CP were higher compared to those of nonusers. This was consistent in individuals aged > 40 years in both genders, especially with long-term use.

## Introduction

Statins are the most widely used lipid-lowering agents for treating hypercholesterolemia and cardiovascular disease. They inhibit 3-hydroxy-3 methylglutaryl-coenzyme A (HMG-CoA) reductase, and statin therapy is generally considered safe and well-tolerated^1^. Statins efficiently suppress low-density lipoprotein cholesterol levels and have pleiotropic effects of systemic anti-inflammatory activity, including local reduction of atherosclerotic plaque formation, enhancement of antimicrobial and fungicidal activities, and bone modulation^1–4^. Considering these benefits, many studies have suggested that statins have supplemental clinical benefits of reducing periodontal inflammation and preventing alveolar bone loss in periodontitis^5,6^. Other studies have reported contradictory results^7–10^.

Chronic periodontitis (CP) is a multifactorial infectious inflammatory disease closely related to host immune system, environmental, and systemic disorder risk factors, including inflammatory disease states, dyslipidemia, cancers, and dysbiosis^11–13^. It is characterized by immune-inflammatory infiltration of the deeper compartments of the periodontium, resulting in destruction of the tooth-supporting tissues such as the cementum, periodontal ligament, and alveolar bone and impaired masticatory function, which culminates in tooth loss and negatively impacts patient quality of life^14,15^. CP is highly prevalent among the elderly, who are often prescribed statins to control cardiovascular disease^16^; this highlights the significance of exploring the association between statin use and periodontitis^17^. Orally administered statins were reported to improve healing outcomes and decrease inflammatory activity in experimental periodontitis in rat models and patients with CP undergoing treatment^2,5,15–18^. Local administration of statins, adjunctive to nonsurgical/surgical periodontal treatment, was also effective in preventing alveolar bone loss in clinical and animal studies of periodontitis^6,18^. Nevertheless, its safety has not been fully elucidated in the short- and long-term regarding CP.

Unexpected events related to the statin use have been reported in the last two decades, raising safety concerns regarding statin therapy^4,19^. The mechanism underlying these observations remains unclear. Recent systematic reviews have reported statins to be safe therapeutic agents for improving oral health^14,20^. Most of previous clinical and animal studies focused on the relatively short-term effects of statins on the severity of pre-existing periodontitis, and had limited sample sizes^6–8,15,18^. Research on the long-term association between statins and CP in large patient cohorts has been scarce. In a previous study^19^, higher healing outcomes was observed in patients with periodontitis on statins for long periods compared with controls; however, the duration of statin intake was not clearly described, and the groups were not age or gender matched. Therefore, longitudinal studies on different populations and with large sample sizes are needed to confirm the relationship between statins and clinical outcomes in CP.

Based on this evidence, we hypothesized that long-term and short-term statin use might have different effects in CP. The null hypothesis is that there is no difference of occurrence of CP between statin users and control (no-statin users). A longitudinal follow-up study using a nationwide, population-based cohort among Korean adults was carried out to estimate the potential impact of statins on periodontitis-related outcomes depending on the duration of statin intake.

## Methods

### Participant selection

This study was approved by the ethics committee of Hallym University (approval number 2019-10-023) and the requirement for written informed consent was waived by the institutional review board. This study was performed in accordance with the relevant guidelines and regulations of the Declaration of Helsinki.

This study was conducted using the Korean National Health Insurance Service-Health Screening (KNHIS-HS) database, which offers deidentified population-based electronic files for research purposes in the Korean population, as previously described^21^. The diagnostic codes of the KNHIS-HS data follow the International Classification of Diseases, 10th Revision, Clinical Modification (ICD-10-CM). From a total of 514,866 individuals with 615,488,428 medical claim codes (from 2002 to 2015), those who first had statin prescriptions for a minimum of 6 months were included in the statin-user group (n = 192,296), while others who had never prescribed statins were included in the control group (n = 322,570). The index date of each statin user was defined as the first date of statin prescription. The index date of the control group was determined as the index date of their matched statin users. We excluded the statin users from 2002 to 2003 (n = 17,670), as we included pre-existing CP before the index date in the analysis. Furthermore, we excluded patients who had incomplete records of cholesterol (n = 68), blood pressure (n = 23), fasting blood glucose (n = 6), or body mass index (BMI, kg/m^2^) (n = 6) from the statin user group. In the control group, we excluded 1474 individuals who either died before 2004 or had no records since 2004, as well as 23,650 patients who received a single prescription of statins from 2002 to 2015.

The statin-user group was matched 1:1 with the control group by propensity-score matching through logistic regression for age, gender, income, and region of residence. To avoid any selection bias, statin nonusers were extracted according to a random number order. During the matching process, 128,065 control group and 5142 statin users were excluded. After exclusion and matching, 169,381 statin users were matched with 169,381 control group non-users, and risk of CP was analyzed as the primary outcome of interest.

**Figure 1:**
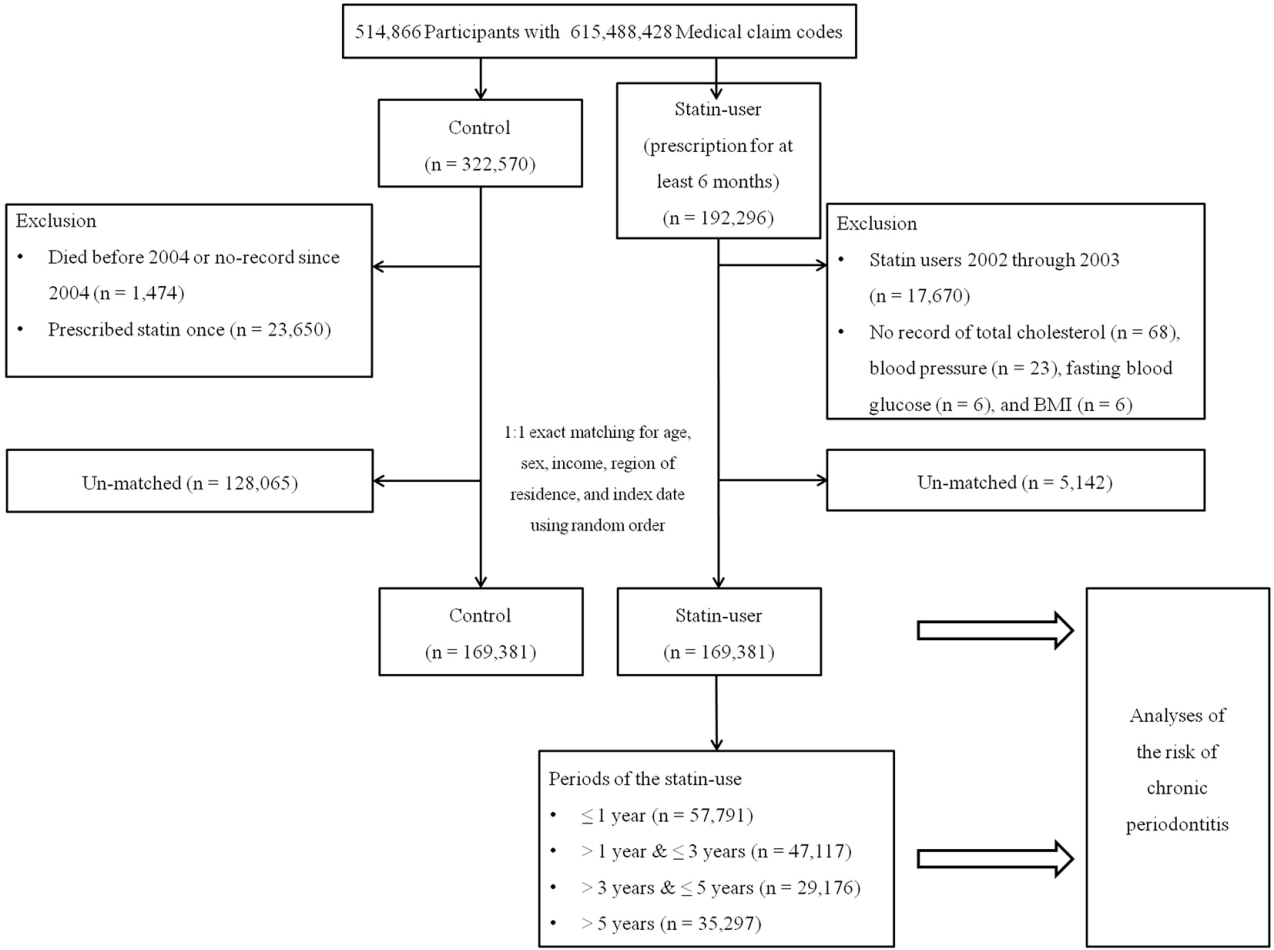
A schematic illustration of the participant selection process that was used in the present study. Of a total of 514,866 participants, 169,381 of the statin-user group were matched with 169,381 of the control group for age, gender, income, and region of residence. Statin-user group was subclassified according to the periods of statin-use as follows: ≤ 1 year (n = 57,791), > 1 year & ≤ 3 years (n = 47,117), > 3 years & ≤ 5 years (n = 29,176), and > 5 years (n = 35,297). Abbreviations: BMI, body mass index.

The statin-user group was subcategorized according to the duration of statin use as follows: ≤ 1 year (n = 57,791), > 1 year to ≤ 3 years (n = 47,117), > 3 years to ≤ 5 years (n = 29,176), and > 5 years (n = 35,297). The risk of CP was analyzed among the different time periods. Figure 1 summarizes the flow illustration of the study population enrollment.

### Independent variable (statin prescription)

Statin prescription was considered the independent variable. The date on which statins were first prescribed was defined as the index date, and subsequent statin use data was analyzed. The statin-user group was defined as patients who were prescribed statins for a minimum of 6 months between 2002 and 2015. The patients deemed short-term users were those who have as 6 months–1 year prescription, as previously described^22^.

### Dependent variable (chronic periodontitis)

CP was considered the dependent variable. The diagnosis of CP according to ICD-10 code (K05.3: Chronic periodontitis) and treatment by dentists was analyzed.

### Covariates

Participants were classified according to age using 10 groups of 5-year intervals, from 40–44 years to > 85 years; according to income into five classes (class 1 [lowest]–class 5 [highest]); and according to regions of residence into urban and rural provinces, based on the previous study^23^. Tobacco smoking, alcohol consumption, and obesity (BMI, kg/m^2^) were subdivided as described in our previous study^23^. The presence of dyslipidemia was defined by at least 1 claim per year for the prescription of an antihyperlipidemic agent under ICD-10 code E78. Fasting blood glucose level (mg/dL), systolic blood pressure (SBP, mmHg), diastolic blood pressure (DBP, mmHg), and total cholesterol level (mg/dL) were obtained. Previously diagnosed CP was defined as having a claim for treatment by dentists due to ICD-10 code (K05.3: Chronic periodontitis) prior to the index^24^. The Charlson Comorbidity Index (CCI) score was calculated, which is widely applied to quantify disease burden using seventeen comorbidities, as the continuous variable (0 [no comorbidities] to 29 [multiple comorbidities])^25^.

### Statistical analyses

Differences between the study and control groups were adjusted through propensity-score matching calculated by logistic regression with the aforementioned four baseline covariates using the greedy option of the nearest-neighbor matching algorithm^26^. After matching, balance between groups was evaluated again. Categorical data were summarized with numbers and percentages. Continuous data were depicted as the mean and standard deviation. Characteristics between the statin user and control groups were compared using the chi-square test for categorical variables and the independent t-test or one-way analysis of variance (ANOVA) for continuous variables.

To analyze the hazard ratio (HR) with 95% confidence interval (CI) for CP (primary outcome of interest) between statin users and control groups, a stratified Cox proportional hazard model was constructed; crude and adjusted models (adjusted for fasting blood glucose, alcohol consumption, obesity, dyslipidemia history, total cholesterol, smoking, SBP, DBP, the number of pre-existing CP, and CCI scores) were also created. The analysis was stratified according to age, gender, areas of residence, and income. For further subgroup analyses, participants were subdivided according to age (< 60 and ≥ 60 years old) and gender (men and women) and both the crude and adjusted models were analyzed.

To analyze the HR with 95% CI for CP among the duration of statin use (secondary objective), an unstratified cox proportional hazard method was performed. In this analysis, crude model 1 (adjusted for fasting blood glucose, CCI scores, obesity, smoking, total cholesterol, alcohol consumption, SBP, and DBP), and model 2 (dyslipidemia history and the number of previous CP diagnoses added to model 1) were calculated.

Statistical analyses were conducted with SAS version 9.4 (SAS Institute., Cary, NC, USA). Two-tailed *P* < 0.05 indicates statistical significance.

## Results

### Baseline characteristics

The study comprised 169,381 patients with history of statin use and an equal number of matched controls, both retrieved from the database. The average duration from January 1, 2002 to CP diagnosis was 454,036 person-years in the statin user group and 484,893 person-years in the control group. Age, region of residence, gender, and income were evenly matched between the two groups (*P* = 1.000; Table 1). The development of new CP during the study period was higher in the statin user group (66.9% [113,349 of 169,381]) than in the control group (62.9% [106,562 of 169,381]) (*P* < 0.001). The rates of dyslipidemia, obesity, smoking habits, comorbidities, frequent alcohol consumption (≥ 1/week), higher total cholesterol or fasting glucose levels, and hypertension were higher in statin users compared to controls (*P* < 0.001).

**Table 1:** General characteristics of participants between statin and control groups.

Characteristics	Total participants		
	Statin	Statin non-user	*P*-value
Age (years old, n, %)			1.000
40–44	1646 (1.0)	1646 (1.0)	
45–49	13,335 (7.9)	13,335 (7.9)	
50–54	33,627 (19.9)	33,627 (19.9)	
55–59	34,815 (20.6)	34,815 (20.6)	
60–64	30,282 (17.9)	30,282 (17.9)	
65–69	24,429 (14.4)	24,429 (14.4)	
70–74	16,780 (9.9)	16,780 (9.9)	
75–79	9485 (5.6)	9485 (5.6)	
80–84	4005 (2.4)	4005 (2.4)	
85+	977 (0.6)	977 (0.6)	
Gender (n, %)			1.000
Males	82,117 (48.5)	82,117 (48.5)	
Females	87,264 (51.5)	87,264 (51.5)	
Income (n, %)			1.000
1 (lowest)	27,596 (16.3)	27,596 (16.3)	
2	22,433 (13.2)	22,433 (13.2)	
3	26,619 (15.7)	26,619 (15.7)	
4	35,917 (21.2)	35,917 (21.2)	
5 (highest)	56,816 (33.5)	56,816 (33.5)	
Region of residence (n, %)			1.000
Urban	74,748 (44.1)	74,748 (44.1)	
Rural	94,633 (55.9)	94,633 (55.9)	
Total cholesterol (mg/dL, mean, SD)	225.6 (40.2)	191.7 (32.5)	< 0.001^†^
SBP (mmHg, mean, SD)	130.0 (17.3)	125.5 (16.7)	< 0.001^†^
DBP (mmHg, mean, SD)	80.2 (11.0)	77.8 (10.7)	< 0.001^†^
Fasting blood glucose (mg/dL, mean, SD)	106.9(36.5)	97.4 (24.8)	< 0.001^†^
Obesity (n, %)^‡^			< 0.001*
Underweight	2115 (1.3)	5494 (3.2)	
Normal	47,435 (28.0)	67,007 (39.6)	
Overweight	47,704 (28.2)	45,855 (27.1)	
Obese I	64,908 (38.3)	47,238 (27.9)	
Obese II	7219 (4.3)	3787 (2.2)	
Smoking status (n, %)			< 0.001*
Nonsmoker	120,170 (71.0)	123,188 (72.7)	
Past smoker	21,082 (12.5)	18,789 (11.1)	
Current smoker	28,129 (16.6)	27,404 (16.2)	
Alcohol consumption (n, %)			< 0.001*
< 1 time a week	114,307 (67.5)	117,201 (69.2)	
≥ 1 time a week	55,074 (32.5)	52,180 (30.8)	
CCI score (score, n, %)			< 0.001*
0	104,801 (61.9)	125,212 (73.9)	
1	29,556 (17.5)	19,495 (11.5)	
2	15,741 (9.3)	11,156 (6.6)	
3	8625 (5.1)	5355 (3.2)	
≥ 4	10,658 (6.3)	8.163 (4.8)	
Dyslipidemia (n, %)	134,151 (79.2)	18,848 (11.1)	< 0.001*
The number of periodontitis before index date (mean, SD)	2.7 (4.5)	2.4 (4.2)	< 0.001^†^
Periodontitis (n, %)	113,349 (66.9)	106,562 (62.9)	< 0.001*

*SBP* systolic blood pressure, *DBP* diastolic blood pressure, *CCI* Charlson comorbidity index, *SD* standard deviation.

*Chi-square test. Significance at *P* < 0.05.

^†^Independent *t* test. Significance at *P* < 0.05.

^‡^Obesity (BMI, body mass index, kg/m^2^) was categorized as < 18.5 (underweight), ≥ 18.5 to < 23 (normal), ≥ 23 to < 25 (overweight), ≥ 25 to < 30 (obese I), and ≥ 30 (obese II).

### Characteristics of statin users according to the duration of use

Women tended to use statins for a longer duration than men (*P* < 0.001) (Table 2). Long-term statin users (> 5 years) tended to be of a higher level of income, from an urban region, obese (BMI 25–30), non-smokers, and less consumptive of alcohol (less than once a week), as well as having more comorbidities than short-term users (≤ 1 year) (*P* < 0.001). Blood pressure, fasting blood glucose level, and total cholesterol level were the highest among those who used statins for > 5 years (all *P* < 0.001). The rate of pre-existing periodontitis decreased with increased duration of statin use (*P* < 0.001), while the incidence of dyslipidemia and subsequent CP tended to increase with an increase in duration (1–3 years, 3–5 years, or > 5 years) compared to short-term duration (≤ 1 year) (each *P* < 0.001).

**Table 2:** General characteristics of participants in statin user according to the periods of the statin-use.

Characteristics	The periods of the statin-use				
	≤ 1 y (n = 57,791)	1–3 y (n = 47,117)	3-5y (n = 29,176)	> 5 y (n = 35,297)	*P*-value
Age (years old, n, %)					< 0.001*
40–44	509 (0.9)	375 (0.8)	246 (0.8)	516 (1.5)	
45–49	3941 (6.8)	3235 (6.9)	2420 (8.3)	3739 (10.6)	
50–54	11,886 (20.6)	9501 (20.2)	5847 (20.0)	6393 (18.1)	
55–59	12,342 (21.4)	9817 (20.8)	5754 (19.7)	6902 (19.6)	
60–64	10,087 (17.5)	8429 (17.9)	5228 (17.9)	6538 (18.5)	
65–69	7480 (12.9)	6756 (14.3)	4416 (15.1)	5777 (16.4)	
70–74	5421 (9.4)	4719 (10.0)	3052 (10.5)	3588 (10.2)	
75–79	3594 (6.2)	2773 (5.9)	1632 (5.6)	1486 (4.2)	
80–84	1959 (3.4)	1213 (2.6)	498 (1.7)	335 (1.0)	
85+	572 (1.0)	299 (0.6)	83 (0.3)	23 (0.1)	
Gender (n, %)					< 0.001*
Males	29,296 (50.7)	22,765 (48.3)	13,467 (46.2)	16,589 (47.0)	
Females	28,495 (49.3)	24,352 (51.7)	15,709 (53.8)	18,708 (53.0)	
Income (n, %)					< 0.001*
1 (lowest)	9621 (16.7)	7721 (16.4)	4766 (16.3)	5488 (15.6)	
2	8061 (14.0)	6328 (13.4)	3722 (12.8)	4322 (12.2)	
3	9284 (16.1)	7509 (15.9)	4521 (15.5)	5305 (15.0)	
4	12,344 (21.4)	10,001 (21.2)	6144 (21.1)	7428 (21.0)	
5 (highest)	18,481 (32.0)	15,558 (33.0)	10,023 (34.4)	12,754 (36.1)	
Region of residence (n, %)					< 0.001*
Urban	24,068 (41.7)	20,359 (43.2)	13,248 (45.4)	17,073 (48.4)	
Rural	33,723 (58.4)	26,758 (56.8)	15,928 (54.6)	18,224 (51.6)	
Total cholesterol (mg/dL, mean, SD)	223.1 (39.7)	224.8 (39.6)	227.4 (39.8)	229.3 (41.6)	< 0.001^†^
SBP (mmHg, mean, SD)	128.1 (16.9)	129.5 (17.0)	130.8 (17.3)	132.9 (17.8)	< 0.001^†^
DBP (mmHg, mean, SD)	79.2 (10.7)	79.9 (10.8)	80.7 (11.1)	82.0 (11.4)	< 0.001^†^
Fasting blood glucose (mg/dL, mean, SD)	104.3 (33.1)	107.1 (36.1)	108.1 (37.0)	110.0 (41.5)	< 0.001^†^
Obesity (n, %)^‡^					< 0.001*
Underweight	984 (1.7)	584 (1.2)	289 (1.0)	258 (0.7)	
Normal	18,126 (31.4)	13,404 (28.5)	7640 (26.2)	8265 (23.4)	
Overweight	16,270 (28.2)	13,361 (28.4)	8202 (28.1)	9871 (28.0)	
Obese I	20,474 (35.4)	17,791 (37.8)	11,651 (39.9)	14,992 (42.5)	
Obese II	1937 (3.4)	1977 (4.2)	1394 (4.8)	1911 (5.4)	
Smoking status (n, %)					< 0.001*
Nonsmoker	39,842 (68.9)	32,980 (70.0)	21,064 (72.2)	26,284 (74.5)	
Past smoker	8098 (14.0)	6335 (13.5)	3461 (11.9)	3188 (9.0)	
Current smoker	9851 (17.1)	7802 (16.6)	4651 (15.9)	5825 (16.5)	
Alcohol consumption (n, %)					< 0.001*
< 1 time a week	34,566 (59.8)	31,649 (67.2)	21,010 (72.0)	27,082 (76.7)	
≥ 1 time a week	23,225 (40.2)	15,468 (32.8)	8166 (28.0)	8215 (23.3)	
CCI score (score, n, %)					< 0.001*
0	37,693 (65.2)	29,712 (63.1)	17,578 (60.3)	19,818 (56.2)	
1	8906 (15.4)	7944 (16.9)	5437 (18.6)	7269 (20.6)	
2	4685 (8.1)	4249 (9.0)	2855 (9.8)	3952 (11.2)	
3	2671 (4.6)	2321 (4.9)	1517 (5.2)	2116 (6.0)	
≥ 4	3836 (6.6)	2891 (6.1)	1789 (6.1)	2142 (6.1)	
Dyslipidemia (n, %)	42,186 (73.0)	38,089 (80.8)	24,189 (82.9)	29,687 (84.1)	< 0.001*
Periodontitis before index date (number, mean, SD)	3.2 (5.1)	3.1 (5.0)	2.4 (3.9)	1.6 (2.8)	< 0.001^†^
Periodontitis (n, %)	32,892 (56.9)	30,812 (65.4)	21,342 (73.2)	28,303 (80.2)	< 0.001*

*SBP* systolic blood pressure, *DBP* diastolic blood pressure, *CCI* Charlson comorbidity index, *SD* standard deviation.

*Chi-square test. Significance at *P* < 0.05.

^†^One-way analysis of variance (ANOVA). Significance at *P* < 0.05.

^‡^Obesity (BMI, body mass index, kg/m^2^) was categorized as < 18.5 (underweight), ≥ 18.5 to < 23 (normal), ≥ 23 to < 25 (overweight), ≥ 25 to < 30 (obese I), and ≥ 30 (obese II).

### Association between statin use and CP

The influence of statin use on the occurrence of new CP was analyzed and compared with that in the control group (Table 3). A higher incidence of newly diagnosed CP was found among statin users than in the controls during the follow-up period (25.0% vs. 22.0% per 100 person-years, *P* < 0.001). Cox regression analysis revealed that patients who used statins had an elevated likelihood of developing new CP compared to the control group after adjusting for demographic data and medical comorbidities, including age, pre-existing CP, history of dyslipidemia, total cholesterol, and fasting blood glucose (HR 1.32; 95% CI 1.30–1.33; *P* < 0.001).

**Table 3:** Hazard ratio (95% confidence interval) for CP in the statin-user and control groups with subgroup analyses according to age and sex.

Characteristics	CP/Total (n)	FU (Person-year)	IR	Hazard ratios for CP			
				Crude^†^	*P*-value	Adjusted^†‡^	*P*-value
**Total participants (n = 338,762)**							
Statin-user	113,349/169,381	454,036	25.0	1.12 (1.11–1.13)	< 0.001*	1.32 (1.30–1.33)	< 0.001*
Control	106,562/169,381	484,893	22.0	1		1	
**Age < 60 years old (n = 166,846)**							
Statin-user	60,305/83,423	230,533	26.2	1.11 (1.10–1.12)	< 0.001*	1.38 (1.36–1.41)	< 0.001*
Control	57,625/83,423	248,067	23.2	1		1	
**Age ≥ 60 years old (n = 171,916)**							
Statin-user	53,044/85,958	223,503	23.7	1.13 (1.12–1.14)	< 0.001*	1.27 (1.25–1.29)	< 0.001*
Control	48,937/85,958	236,826	20.7	1		1	
**Males (n = 164,234)**							
Statin-user	55,755/82,117	195,398	28.5	1.13 (1.11–1.14)	< 0.001*	1.29 (1.26–1.31)	< 0.001*
Control	52,477/82,117	210,721	26.5	1		1	
**Females (n = 174,528)**							
Statin-user	57,594/87,264	258,638	22.3	1.11 (1.10–1.13)	< 0.001*	1.35 (1.33–1.38)	< 0.001*
Control	54,085/87,264	274,172	19.7	1		1	

*CP* chronic periodontitis, *FU* Follow-up duration, *IR* Incidence rate per 100 person-years.

*Stratified Cox proportional hazard model, Significance at *P* < 0.05.

^†^Models were stratified by age, gender, income, and region of residence.

^‡^Adjusted for dyslipidemia history, total cholesterol, systolic blood pressure, diastolic blood pressure, fasting blood glucose, obesity, smoking, alcohol consumption, the number of previous CP, and CCI scores.

The incidence of CP during the study period was higher in statin users than in the control group in all age and gender subgroups. In subgroup analyses, statin use was consistently associated with a high likelihood of having subsequent CP among those aged either < 60 years or ≥ 60 years ([HR 1.38; 95% CI 1.36–1.41; *P* < 0.001] and [HR 1.27; 95% CI 1.25–1.29; *P* < 0.001], respectively) and among both men and women ([HR 1.29; 95% CI 1.26–1.31; *P* < 0.001] and [HR 1.35; 95% CI 1.33–1.38; *P* < 0.001], respectively).

### Association between duration of statin use and CP

The participants with either 1–3 years, 3–5 years, or > 5 years of statin use demonstrated higher HR for CP compared with patients who used statins for ≤ 1 year (1.08 [95% CI 1.07–1.10, *P* < 0.001]; 1.08 [95% CI 1.06–1.09, *P* < 0.001]; 1.04 [95% CI 1.02–1.06, *P* < 0.001], respectively) (Table 4). However, the HRs were slightly reduced in the group with > 5 years of statin use compared to durations of 3–5 years or 1–3 years (*P* for trend = 0.002).

**Table 4:** Hazard ratio (95% confidence interval) for CP according to the periods of statin-use (n = 169,381).

Periods of statin-use	CP/Total (n)	FU (Person-year)	IR	Hazard ratios for CP					
				Crude	*P*-value	Model 1^†^	*P*-value	Model 2^‡^	*P*-value
*P* for trend					< 0.001*		< 0.001*		0.002*
> 5 year	28,303/35,297	129,503	21.9	0.94 (0.92–0.95)	< 0.001*	0.96 (0.95–0.98)	< 0.001*	1.04 (1.02–1.06)	< 0.001*
3–5 year	21,342/29,176	86,144	24.8	1.01 (0.99–1.03)	0.310	1.03 (1.01–1.05)	0.003*	1.08 (1.06–1.09)	< 0.001*
1–3 year	30,812/47,117	113,073	27.2	1.06 (1.04–1.07)	< 0.001*	1.07 (1.05–1.09)	< 0.001*	1.08 (1.07–1.10)	< 0.001*
≤ 1 year (Ref)	32,892/57,791	125,316	26.2	1		1		1	

*FU* Follow-up duration, *IR* Incidence rate per 100 person-years, *CCI* Charlson comorbidity index, *CP* chronic periodontitis.

*Un-stratified Cox proportional hazard model, Significance at *P* < 0.05.

^†^A model 1 was adjusted for total cholesterol, systolic blood pressure, diastolic blood pressure, fasting blood glucose, obesity, smoking, alcohol consumption, and CCI scores.

^‡^A model 2 was adjusted for model 1 plus dyslipidemia history and the number of previous CP.

## Discussion

We found the difference of CP between statin user and control with statistical significance. It was evident in both men and women aged over 40 years. Among long-term statin users, pre-existing periodontitis was low while newly occurring periodontitis was high. The relation between statin use and increased likelihood of CP occurrence remained significant even after adjustment for confounders, indicating that statin use was independently associated with increased risk of developing CP.

Large-scale nationwide studies that examine the relationship between statin use and CP risk based on duration of use are scarce. Among the 338,762 participants aged ≥ 40 years, those receiving statins exhibited a 1.32-fold higher chance of developing CP (95% CI1.30–1.33) than the matched control participants (incidence: 25.0% and 22.0% per 100 person-years, respectively). Because the prevalence of periodontitis increases with age^27^, we adjusted for age in the analysis; the association between statin use and likelihood of developing new CP (HR 1.32; 95% CI 1.30–1.33) persisted. Although the magnitude of risk is low, statin use may slightly increase the risk of developing CP. Long-term statin users (1–3 years, 3–5 years, or > 5 years) had a higher risk for CP than short-term users (≤ 1 year). Pre-existing periodontitis decreased in long-term statin users compared to short-term users, implying that statins may likely be effective in decreasing pre-existing periodontitis but might increase the chance of developing newly occurring periodontitis. The decrease in pre-existing CP during the 5-year period is partly in line with results from a prospective double-blind, randomized study among 83 participants conducted in the USA, which demonstrated that 12 weeks of statin therapy markedly reduced periodontitis^5^. The trend of reduction in periodontitis was perceived in the first 28 days after the initiation of statin intake^5^. An epidemiologic study reported that the use of any statin is associated with a 48% decrease in CP-related tooth loss in the initial three years, which may suggest that statins possess anti-inflammatory and bone modulating features during the first several years that may positively influence pre-existing CP^28^.

Conversely, reports from the literature which support or contradict our findings of increased risk of CP occurrence due to statin therapy among long-term users is scarce. Most of the previous studies focused on the relatively short-term effects of statins on the inflammatory parameters of pre-existing periodontitis. The accuracy of the present data is supported by the similar clinical characteristics of statin users with respect to obesity, smoking, increased comorbidities, alcohol drinking, or dyslipidemia, and high total cholesterol, blood pressure, or fasting glucose level, as reported in previous studies^1,4,5,16,19^. We further found that long-term statin users are more likely to be of a higher income level, from an urban region, obese, non-smokers, and less consumptive of alcohol, as well as having more comorbidities, than short-term users. This might be because of the pleiotropic effects (whatever adverse or beneficial) reported in statin use among coronary disease patients, even with average serum cholesterol levels^29,30^, which implies that statins exert a wider range of adverse or beneficial effects independent of their regulation on cholesterol or coronary artery disease^4,27–29^. In a previous study^2^, it was mentioned that 15 subsequent periodontitis cases among 29 patients with hyperlipidemia treated with statins were observed during a statin intake period of 3 to 132 months; although the observed duration varied widely, the study could be said to have examined the long-term effects of statins use. Notably, a preliminary study identified a high percentage of oral symptoms associated with statin use; these symptoms markedly improved after suspension of the treatment^9^, raising the possibility of diverse statin-induced oral adverse effects.

Myopathy, rhabdomyolysis, diabetes, and cancers have been reported as adverse effects of statins^4,27–29^. An association between statin-induced adverse events and statin therapy is infrequent^4,27–29^. Clinical trials of pravastatin therapy for hypercholesterolemia with a 15-year follow-up reported a greater incidence of prostate cancer in these patients^1^, which indicates that adverse effects may become evident after a long time, such as a decade or more. Likewise, the incidence of CP related to statin use might have been underestimated because of the short periods of previous studies. Our findings may be of importance with respect to the safety of the long-term use of statins in the development of periodontitis. Therefore, patients who are prescribed statin therapy need to be informed of the increased risk of diseases, including CP.

The mechanism underlying the association between statin use and increased risk of CP remains unclear. Statins have cholesterol-independent or pleiotropic effects attributable to several mechanisms vital to cellular functions via the posttranscriptional modification of mevalonate intermediates in multiple tissues, including the periodontium^1–4,20,31,32^. In fact, the systemic administration of statins has potential effects in the periodontium, and it has been reported that their concentration in gingival crevicular fluid is 10- to 100-fold higher than in the serum, with an anti-inflammatory effect that influences the level of IL-1β in the gingiva. As statins also possess immunomodulatory and antimicrobial properties^33^, their long-term effects might include a shift in the microbial balance between pathogenic and nonpathogenic species in the oral cavity, which has over 700 microorganisms; this deteriorates the periodontal microbiome and causes dysregulation of the immune response of the host^13,32^. Interactions between polymicrobial synergistic and dysbiotic action, host response, and modifying factors may determine the defense against CP or its progression^15^. Individuals with defective neutrophil recruitment or neutrophil adhesion deficiencies have increased susceptibility to periodontitis^34,35^. The range of drug action observed in statin therapy seems to be greater than expected and the precise predictions of adverse events are not possible until those events occur^36^.

The strengths of this study are its large, representative, nationwide population-based data and analysis that was fully adjusted for socioeconomic status, potential risk factors, and comorbidities related to CP or statin use (e.g., fasting blood glucose, total cholesterol, obesity, alcohol, smoking, and blood pressure). To the best of our knowledge, a nationwide follow-up epidemiologic study on the association between long-term statin intake and CP risk has not previously attempted. As the KNHIS-HS data encompasses information from every hospital and clinic across the entire nation without exception, full medical histories could be obtained during the follow-up period.

Our study has some limitations. First, we did not indicate which type of statin patients used, which prevents us from reporting the association between individual statins and CP. This is especially important considering the contradictory results reported in the literature; some statins might be associated with a high or lower chance of developing CP. Second, patient compliance with medication could not be confirmed using the KNHIS-HSC data. Third, no information pertaining to the severity of periodontitis, such as probing depth and clinical attachment loss at interproximal sites; type, dosage, and frequency of statins; and family history and genetic data of related systemic diseases, were available in the health insurance database. Fourth, as this study is not a randomized controlled study, the findings of our study should be interpreted carefully in that our results might have the possibility of being biased from unconsidered covariates. Lastly, the possibility of missing data was not considered in our analysis.

In summary, we found a positive association between statin use and development of CP in the adult Korean population. Thus, we suggest the careful prescription of statins, especially for long-term use, in patients at high risk for developing or in case of CP.

## Data Availability

Releasing of the data by the researcher is not allowed legally. All data are available from the database of National Health Insurance Sharing Service (NHISS) https://nhiss.nhis.or.kr/ NHISS allows access to all of this data for the any researcher who promises to follow the research ethics at some cost. If you want to access the data of this article, you can download it from the website after promising to follow the research ethics.
